# Potassium and Magnesium Mediate the Light and CO_2_ Photosynthetic Responses of Grapevines

**DOI:** 10.3390/biology9070144

**Published:** 2020-06-28

**Authors:** Suzy Y. Rogiers, Dennis H. Greer, Francesca J. Moroni, Tintu Baby

**Affiliations:** 1 NSW Department of Primary Industries, Wagga Wagga, NSW 2678, Australia; 2 National Wine and Grape Industry Centre, Charles Sturt University, Wagga Wagga, NSW 2678, Australia; dennisgreer84@gmail.com (D.H.G.); fmoroni@csu.edu.au (F.J.M.); tbaby@csu.edu.au (T.B.)

**Keywords:** A-ci response curves, nutrient deficiency, photosynthesis, *Vitis vinifera*

## Abstract

Potassium (K) and magnesium (Mg) deficiency are common stresses that can impact on grape yield and quality, but their effects on photosynthesis have received little attention. Understanding the diffusional and biochemical limitations to photosynthetic constraints will help to guide improvements in cultural practices. Accordingly, the photosynthetic response of *Vitis vinifera* cvs. Shiraz and Chardonnay to K or Mg deficiency was assessed under hydroponic conditions using miniature low-nutrient-reserve vines. Photosynthesis was at least partly reduced by a decline in stomatal conductance. Light and CO_2_-saturated photosynthesis, maximum rate of ribulose 1.5 bisphospate (RuBP) carboxylation (*V_cmax_*) and maximum rate of electron transport (*J_max_*) all decreased under K and Mg deficiency. Likewise, chlorophyll fluorescence and electron transport were lower under both nutrient deficiencies while dark respiration increased. K deficiency drastically reduced shoot biomass in both cultivars, while root biomass was greatly reduced under both Mg and K deficiency. Taken together, these results indicate that the decrease in biomass was likely due to both stomatal and biochemical limitations in photosynthesis. Optimising photosynthesis through adequate nutrition will thus support increases in biomass with carry-on positive effects on crop yields.

## 1. Introduction

Nutrients are critical for optimum plant growth and development. In grapevines, deficiency of a particular nutrient can alter canopy development, root functioning, flowering, fruit set, ripening and final fruit composition, and thus yield and wine style [1,2,3,4,5]. The beautiful red soils of many grapevine-growing regions in Australia are aged and naturally nutrient-deficient. Even though grapevines are moderate in their nutrient requirements, nutrient starvation can also occur through leaching, run-off, immobilization by clay particles or long-term crop removal. Organic or manufactured fertiliser application for optimal vine performance is a common practise, however, nutrient availability and uptake is influenced by other physical and structural characteristics of the soil, including pH and water content. Consumers and legislators are putting increased pressure for sustainable production practises including the better management of fertilisers [6]. Vine nutrient requirements for optimal crop quality without excess vegetative vigour have received considerable attention, however, there is surprisingly little information on nutrient requirements for the ideal metabolic and physiological performance to help achieve this goal.

Potassium (K) is an essential element that drives growth, maintains turgor and is involved in phloem transport [7,8]. It regulates the cation–anion balance within cells, cytoplasmic pH, membrane potential and activates enzymes [9,10,11]. Potassium is the predominant cation of grape berries and is important to its sugar:acid balance and colour [12,13,14,15]. Moreover, this element is vital to photosynthesis. Potassium promotes chlorophyll synthesis [16] and assimilates export from leaves [17,18]. It is well established that K regulates stomatal behaviour [19,20], and because stomatal closure leads to a barrier in CO_2_ diffusion, it has been demonstrated in several crop plants that leaf net assimilation is curtailed through a decrease in stomatal conductance under K deficiency [21]. Nonstomatal limitations in photosynthesis have also been demonstrated in species such as *Carya cathayensis* Sarg. [22] and *Brassica napus* [23] grown under limited K. The maximum rates of carboxylation (*V_cmax_*) and the regeneration of the carboxylating substrate ribulose 1,5-bisphosphate (RuBP) directed by the electron transport capacity (*J_max_*) are reduced in grapevines exposed to heat and drought [24] and in other species [25], however, how these parameters are affected by nutrient deficiencies is much less understood.

Magnesium (Mg) has many fundamental roles in plant metabolism [26,27]. This macro-element regulates cellular pH and the cation–ion balance and acts as a co-factor for enzymes involved in the formation of DNA and RNA, respiration, N assimilation, transport proteins and photosynthesis, including the protein rubisco [28,29,30]. A substantial proportion of a plant’s Mg is bound up in leaves as it is an essential component of chlorophyll *a/b* in the light harvesting complexes [31]. Magnesium has other roles associated with photosynthesis that are related to charge and membrane mobility [32] and is involved in energy transfer via adenosine triphosphate [33]. Similar to K, Mg deficiency can result in the accumulation of sucrose and starch in leaves through an inhibition of phloem loading [28,30,34]. From a viticultural perspective, Mg may help avoid bunch stem necrosis [35,36] and protect anthocyanins from catabolism in cell vacuoles [37]. Relative to K, berries are not a strong sink for Mg and most of it is partitioned to the roots and vegetative components [38]. Magnesium deficiency can occur in vines growing in sandy, acidic soils as well as in calcareous soils with high pH [39] due to competition with other cations. Even though grapevines are a model for perennial horticultural crops, the impact of Mg deficiency on photosynthesis in grapevines has received limited attention (with the exception of [40]).

The relative importance of diffusional and biochemical limitations to photosynthetic constraints during K or Mg deficiency requires clarification in grapevines to ensure more sustainable uses of these nutrients. In order to avoid the complexity of the soil–vine system, Shiraz and Chardonnay vines were grown hydroponically in Mg or K deficiency and leaf stomatal conductance, and photosynthetic capacity and biomass accumulation were monitored. Mature grapevines store ample nutrient reserves within the perennial woody components [41], and thus a nutrient deficiency response may not be apparent for some time. We, therefore, used miniature vines grown from the small rooted canes in this study to overcome this limitation.

## 2. Materials and Methods

### 2.1. Plant Growth Conditions

This study was undertaken at the National Wine and Grape Industry Centre plant growth facilities at Charles Sturt University in the Riverina, NSW, Australia. After five weeks of rooting, one-year-old dormant cuttings of *Vitis vinifera* cv. Chardonnay and cv. Shiraz vines were transferred into 2.5 L pots containing perlite and established in greenhouse conditions under natural light with an average air temperature of 25 °C during the day and 15 °C during the night. The vines were placed in a randomised block design across three tables, with each table supporting three vines of each treatment. Bud break occurred in these vines in September. Two shoots were retained on each plant and staked vertically.

### 2.2. Nutrient Treatments

Nutrient treatments were made up in separate tanks and pumped to the pots along separate fertigation lines. Fertigation was initiated 1 week prior to budbreak and occurred daily to the point of run-off, where one was a full nutrient treatment (control) based on modified half-strength Hoagland’s solution [42] and the others were the same nutrients, except, in one treatment, potassium was eliminated (K-deficient) and in the remaining treatment, magnesium was eliminated (Mg-deficient). The nitrate and phosphate concentrations were equal across the treatments. The Mg and K content of dried petiole samples collected at the 10-leaf stage from eight replicate plants were assessed by ICP-OES at a commercial diagnostic lab (Charles Sturt University, Wagga Wagga, NSW, Australia) and presented in Table 1. Mg uptake was greater in the K-deficient vines, as previously reported in similar studies [21,43], but still in the adequate range [44]. N was determined on a 50-mg sample with a VarioMAX combustion analyser (Elementar, Hanau, Germany). All other nutrients were in the adequate range and averaged at 33.4 N, 36.1 Ca, 3.25 P, 1.65 S, 0.018 B, 0.011 Cu, 0.047 Fe, 0.085 Mn, 0.08 Mo and 0.042 g kg^−1^ Zn.

### 2.3. Gas Exchange Measurements

All gas exchange measurements were undertaken with the LiCor LI-6400 XT system (Li-Cor Biosciences, Lincoln, Nebraska) in one configuration with the standard LED lighting system (LI-6400-02B) and the other configuration with the LI-6400-40 fluorometer attached to the cuvette. In all cases, the leaf temperature was maintained at 25 °C, the photon flux density (PFD) was set at 1500 µmol m^−2^ s^−1^ except when varied in the light responses and the CO_2_ concertation was maintained at 400 µmol mol^−1^. The leaf-air vapour pressure deficit was not controlled but remained at about 1.5 kPa. The red to blue ratio was maintained at 9:1 in both the standard LED lighting system and the fluorometer. The area of illumination was 2 cm^2^.

### 2.4. Gas Exchange Along the Shoot

At about mid-season, when the shoots on each vine had up to 15 leaves, for each treatment, the gas exchange was measured on every leaf of the randomly selected vines of both cultivars, at least until the leaves were too small to fully cover the chamber. All measurements were undertaken as above on three vines per treatment for each cultivar. The most basal bud was not counted as a node.

### 2.5. Photosynthetic Responses to Light

Gas exchange was measured at the constant conditions above on the youngest fully expanded leaves on shoots of randomly chosen vines in each treatment. For each light response, the PFD was initially set at 1500 µmol m^−2^ s^−1^ and, when the rates were steady, the PFD was decreased progressively in selected steps until approximately 1 µmol m^−2^ s^−1^ (dark). The procedure was repeated 2–3 times for each treatment.

### 2.6. Photosynthetic Responses to Internal CO_2_

On each occasion, fully expanded leaves were used and the gas exchange conditions were as above. For each *A/ci* response, the reference CO_2_ concentration was set at 400 µmol mol^−1^ until the rates were steady, and then the CO_2_ concentration was reduced in 50–100 µmol mol^−1^ steps to 50 µmol mol^−1^. Thereafter the CO_2_ concentration was increased back to 400 µmol mol^−1^ to ensure that rates had returned close to the initial rates, and then the CO_2_ concentration was increased in 100–200 µmol mol^−1^ to 1600 µmol mol^−1^. Each response was determined for leaves on each of the nutrient treatments and replicated 3–4 times.

Simultaneous chlorophyll fluorescence was measured during each of the *A/ci* responses. Throughout each response and at each change in CO_2_ concentration, the steady-state fluorescence in the light (*F_s_*) and the maximal fluorescence in the light (*F_m_’*) were measured. Then, the actinic light was turned off briefly and low-intensity far-red light was turned on for 3 s to enable measurement of the light-adapted minimal fluorescence (*F_o_’*) to be measured.

### 2.7. Chlorophyll Index

The chlorophyll index (CI) was measured of leaves on node position 8 from the shoot base with a chlorophyll meter (SPAD-502, Minolta, Osaka, Japan). The SPAD measurement per leaf consisted of an average of three readings (one reading per apical leaf lobe and both lateral leaf lobes).

### 2.8. Biomass

Twelve weeks after budburst, the vines were dismembered into roots and shoots (stems and leaves). The original cane from which the roots and shoots originated was omitted from the biomass assessment. The vine components were dried in a forced-air oven at 60 °C and dry weight was assessed once stable.

### 2.9. Data Analysis

All data were analysed using a general linear model (GLM) approach using SAS 9.1.3 (SAS Institute Inc., Cary, NC, USA) and least squares means and standard errors were determined assuming a fully randomised experimental design. The photosynthetic light responses were analysed using non-linear regression with SAS to fit a hyperbolic tangent function [45].

For the fitting of the *A/ci* data to the C3 photosynthetic model of Farquhar et al. [46], the apparent maximum rates of RuBP carboxylation (*V_cmax_*) and the apparent maximum rates of RuBP regeneration (*J_max_*) were determined following the procedure of Greer and Weedon [24]. The temperature dependencies of *V_cmax_* and *J_max_* were adopted from Sharkey et al. [47] and all data were analysed using non-linear regressions with SAS. For the determination of the mesophyll conductance (*g_m_*) to calculate chloroplast CO_2_ concentration, the procedures of Pons et al. [48] and Flexas et al. [49], using the electron transport rate estimated from the simultaneous chlorophyll fluorescence measurements, were adopted.

## 3. Results

### 3.1. Photosynthesis Along the Shoot

**Figure 1:**
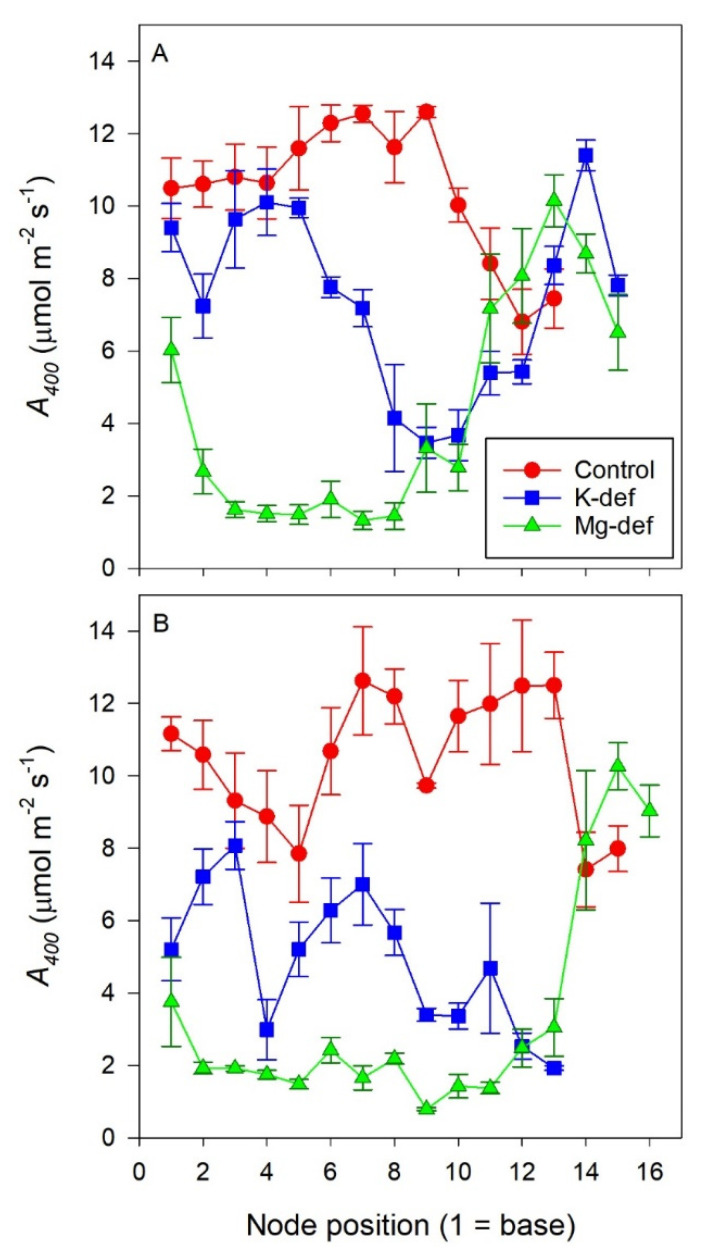
*A_net_* of Chardonnay (**A**) and Shiraz (**B**) leaves with node position along the shoot for vines grown hydroponically in greenhouse conditions in control, K-deficient and Mg-deficient nutrient solutions.

Node position along the stem had a significant impact on leaf *A_net_* in both cultivars (*p* < 0.01) with nutrient deficiency resulting in the lowest values for those leaves located along the middle of the shoot (*p* < 0.01) (Figure 1). Potassium deficiency decreased *A_net_* by up to 50% from nodes 1 to 5 in Chardonnay, however, this was not apparent in Shiraz. Middle-aged Shiraz leaves, between nodes 6−10, were negatively affected by K deficiency, with 30–75% lower *A_net_* relative to the control. In Chardonnay, these middle-aged leaves had about 40–80% reduction in *A_net_*. The assimilation of youngest leaves of the K-deficient vines (node 12 onwards for Shiraz) was not different from the control. In the Mg-deficient vines, *A_net_* was 80% lower in both old and middle-aged leaves, but the younger leaves from node 11 (Shiraz) or 14 (Chardonnay) onwards were not different from the control.

### 3.2. Photosynthetic Light Responses

**Figure 2:**
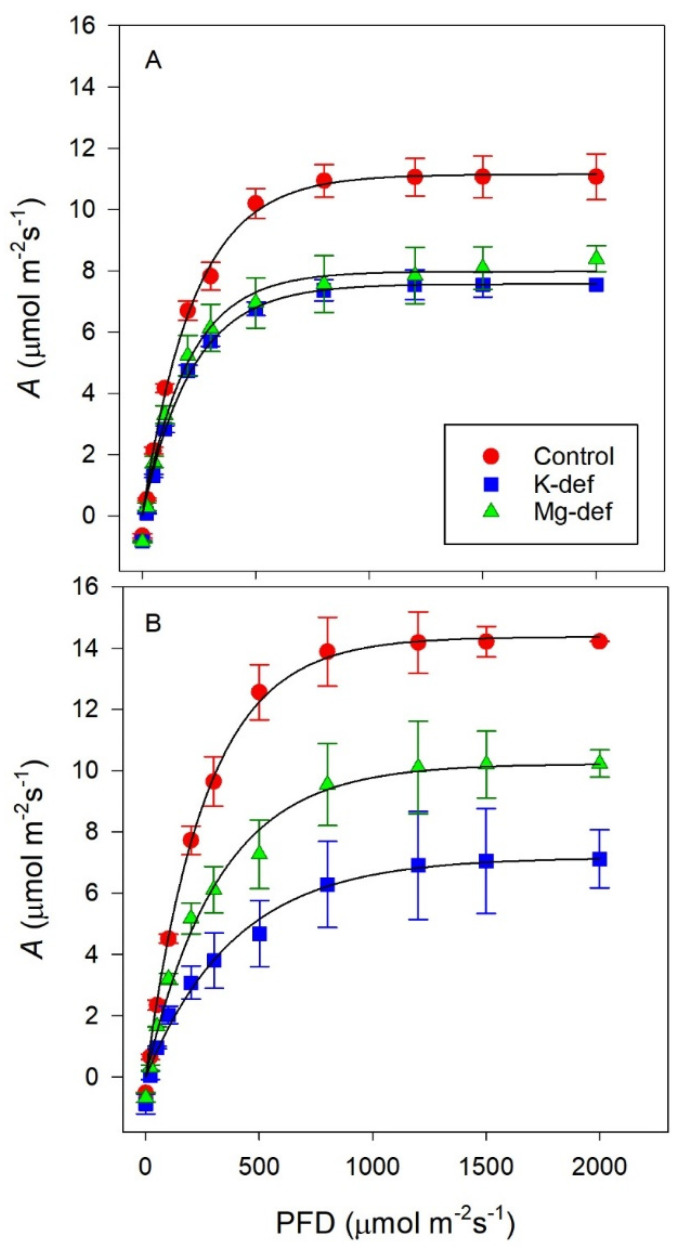
Light response curves for Shiraz (**A**) and Chardonnay (**B**) vines grown hydroponically in greenhouse conditions in control, K-deficient and Mg-deficient nutrient solutions.

The photosynthetic light responses (Figure 2A) indicated that the K and Mg nutrient deficiencies in the Shiraz vines reduced light saturated photosynthesis by similar amounts compared to the control vines. This is shown more generally in Table 1, where the analyses of the fitting of the hyperbolic tangent function to the light responses are shown. However, respiration rates were higher in the nutrient deficient vines, while the PFD at saturation was statistically lower compared to the control vines. Although statistically different, the apparent photon yields were largely unaffected by the nutrient deficiency.

By contrast for the Chardonnay vines (Table 2), the potassium deficiency reduced photosynthesis, especially *A_max_*, much more than occurred in the magnesium-deficient treatment. Compared to the control vines, however, those vines with reduced nutrients had significantly lower light-saturated photosynthetic rates *(A_sat_)*, were light-saturated at higher PFDs but had no significant differences in respiration rates (*R_d_*). The efficiency of photosynthesis was determined by the reduced apparent photon yield (*ϕ_I_*) in the nutrient-deficient vines.

### 3.3. Photosynthetic Response to Chloroplast CO_2_

**Figure 3:**
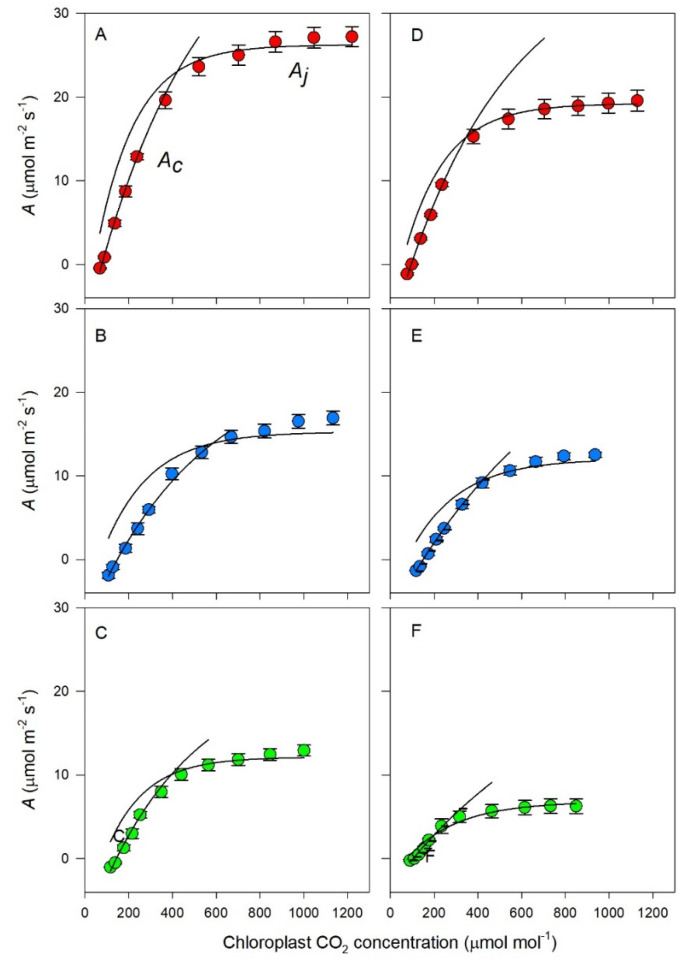
Rates of light-saturated photosynthesis (mean ± s.e.) of leaves as a function of the chloroplast CO_2_ concentration. Shiraz (**A**–**C**) and Chardonnay (**D**–**F**) vines grown hydroponically in greenhouse conditions under control (**A**,**D**), K-deficient (**B**,**C**) or Mg-deficient (**C**,**D**) conditions. The lines fitted to these data are from the C3 model of photosynthesis according to Farquhar et al. (1980). *A_j_* = RuBP regeneration-limited photosynthesis, *A_c_* = Rubisco-limited photosynthesis

The fitting of the C3 photosynthesis model to the photosynthetic response to chloroplast CO_2_ concentration of the control and treated Shiraz and Chardonnay vines (Figure 3) was highly significant (*p* < 0.001; r^2^ = 0.95 − 0.98). For the Shiraz vines, the carboxylation of RuBP was clearly limiting assimilation below about 400 µmol mol^−1^ for each treatment, and regeneration of RuBP was limiting at the higher CO_2_ concentrations. Notably, the response to CO_2_ became progressively diminished from the control to the K-deficient vines to the Mg-deficient vines, with a clear reduction in the CO_2_-saturated assimilation rates. A similar pattern occurred with the Chardonnay vines except that, for all treatments, the CO_2_-saturated assimilation rates were markedly lower compared with those for Shiraz. It was also apparent for the Mg-deficient Chardonnay vines that at low CO_2_ there was co-limitation by RuBP carboxylation and regeneration, but the assimilation rates were markedly reduced.

**Figure 4:**
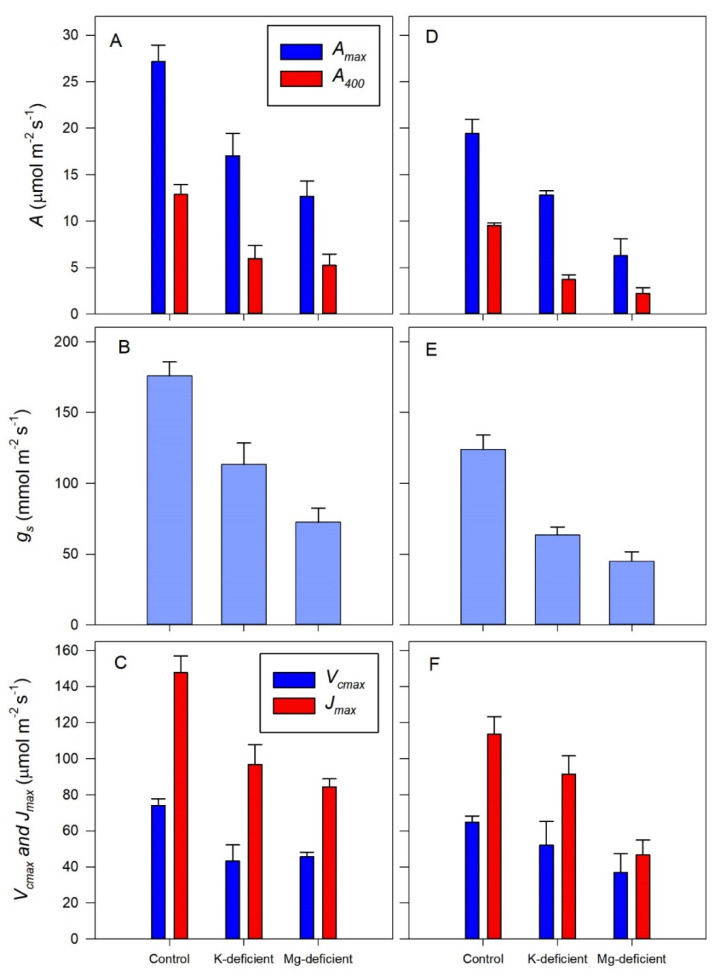
Leaf net assimilation (*A_net_*) (**A**,**D**), stomatal conductance (*g_s_*) (**B**,**E**) and maximum rates of ribulose 1,5-bisphosphate carboxylation (*V_cmax_*) and electron transport (*J_max_*) (**C**,**F**) of grapevines grown hydroponically in greenhouse conditions in control, K-deficient or Mg-deficient nutrient conditions (mean ± s.e.).

The effect of the nutrient deficiency on the *A/c_c_* (assimilation as a function of chloroplast CO_2_ concentration) responses is shown more generally in Figure 4 where the light and CO_2_-saturated assimilation rates (*A_max_*) and the assimilation rates at ambient (400 µmol mol^−1^) CO_2_ concentrations (*A*_400_) for both grapevine cultivars declined markedly. For example, *A_max_* declined by 37% with the K-deficient Shiraz vines and by another 30% for the Mg-deficient vines, compared to the control vines. Comparable reductions in *A_max_* were 37 and 50% for the Chardonnay vines. Thus, a comparable reduction in *A_max_* occurred with K deficiency in both cultivars, but Chardonnay assimilation was much more sensitive to Mg deficiency than Shiraz assimilation.

When CO_2_ was limiting assimilation (*A*_400_), the effects of the nutrient deficiencies were much greater than for *A_max_*, where a 53% reduction occurred for the Shiraz vines and a 61% reduction occurred with the Chardonnay vines in the K-deficient vines compared to the control vines. However, the Mg deficiency caused only a slightly greater reduction in *A*_400_, more in Chardonnay than in Shiraz, compared to the controls. It was apparent that Chardonnay assimilation was again more sensitive to the Mg deficiency, as the rates were negligible.

In part, the changes in assimilation induced by nutrient deficiency were correlated with a similar impact on stomatal conductance (Figure 4B,E) in both cultivars. However, the Shiraz vines had intrinsically more open stomata than the Chardonnay vines, for example, the control Shiraz vines had average stomatal conductance of 176 ± 10 mmol m^−2^ s^−1^ compared to the control Chardonnay vines with an average conductance of 99 ± 8 mmol m^−2^ s^−1^, which is statistically (*p* < 0.01) lower. For the K deficiency, stomatal conductance declined by 35 and 51% compared to the control vines but by a further 36 and 70% reduction for the Mg-deficient vines. Thus, stomata of these Chardonnay vines with Mg–deficiency were for all intents closed, matching the almost negligible assimilation.

Consistent with the cultivar differences in assimilation, the apparent maximum rates of RuBP carboxylation (*V_cmax_*) also differed significantly (*p* < 0.05), from 74.1 ± 3.6 µmol m^−2^ s^−1^ in Shiraz leaves to 64.8 ± 3.4 µmol m^−2^ s^−1^ in the Chardonnay leaves. However, the Chardonnay vines with K deficiency had only a 19% reduction in carboxylation rates compared to 41% reduction for the Shiraz vines. By contrast, Mg deficiency had no more effect on the maximum rates of RuBP carboxylation than the K deficiency in the Shiraz leaves, whereas the rates of carboxylation declined by a further 28% with Mg deficiency in the Chardonnay leaves.

A generally similar response occurred with the apparent maximum rates of RuBP regeneration (*J_max_*), again with statistically (*p* < 0.05) higher rates in the control Shiraz leaves (148 ± 9 µmol m^−2^ s^−1^) compared to the control Chardonnay leaves (113 ± 9 µmol m^−2^ s^−1^). As with *V_cmax_*, the K deficiency had a greater impact on rates of RuBP regeneration (34% reduction) in the Shiraz leaves compared to the Chardonnay leaves (19%) and the Mg deficiency was also comparable, with 13% lower RuBP regeneration rates in the Shiraz leaves and 49% lower rates in the Chardonnay leaves.

### 3.4. Chlorophyll Fluorescence Responses

**Figure 5:**
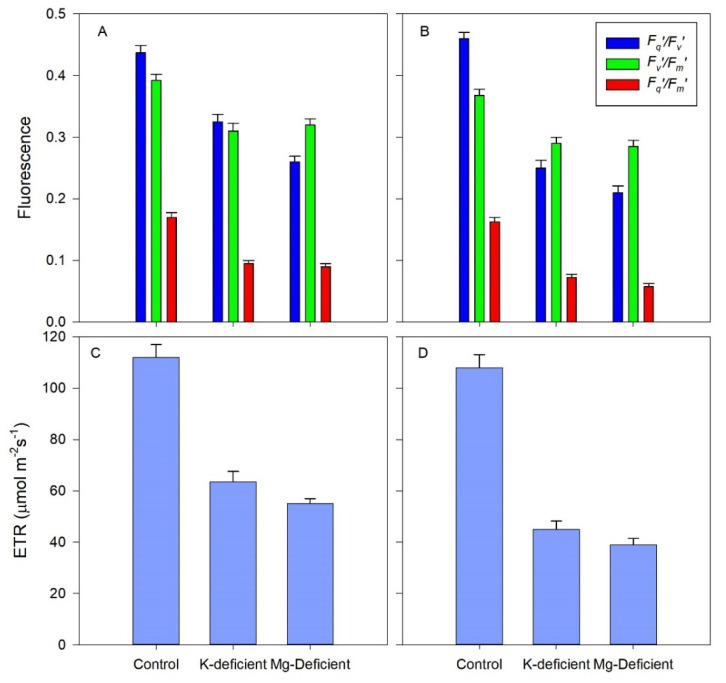
Fluorescence attributes and electron transport rate (ETR) of grapevine leaves grown hydroponically in greenhouse conditions and grown in control, K-deficient or Mg-deficient nutrient solutions. Photochemical quenching (*F_q_′/F_v_′*), maximum efficiency of PSII photochemistry in the light if all PSII centres were open (*F_v_′/F_m_*′), and PSII operating efficiency in the light (*F_q_′/F_m_′*) in Shiraz (**A**) and Chardonnay (**B**). ETR in Shiraz (**C**) or Chardonnay (**D**).

The maximum efficiency of PSII in the light-adapted state (*F_v_′/F_m_′*) did not differ markedly between the two nutrient deficiencies (Figure 5) but was 20–24% lower than the control vines of both cultivars. The actual quantum efficiency of PSII electron transport (*F_q_′/F_m_′*) was highest in the control vines but was reduced in both K-deficient and Mg-deficient vines by 27 and 41% in the Shiraz vines and by 56 and 65% in the Chardonnay vines, thus nutrient deficiencies had a greater effect on Chardonnay photochemistry than on the Shiraz vines. Photochemical quenching (*F_q_′/F_v_′*) increased by about 45% in the K-deficient vines for both cultivars, and a further 10% increase occurred for the Mg-deficient vines, but there were few differences in photochemical quenching between the cultivars. ETR declined in the leaves with the absence of K and Mg nutrients, by 45 and 52%, respectively, in the Shiraz vines and by 56 and 65% in the Chardonnay vines, consistent with the lower photochemical efficiency in the Chardonnay vines.

### 3.5. Chlorophyll Index

**Figure 6:**
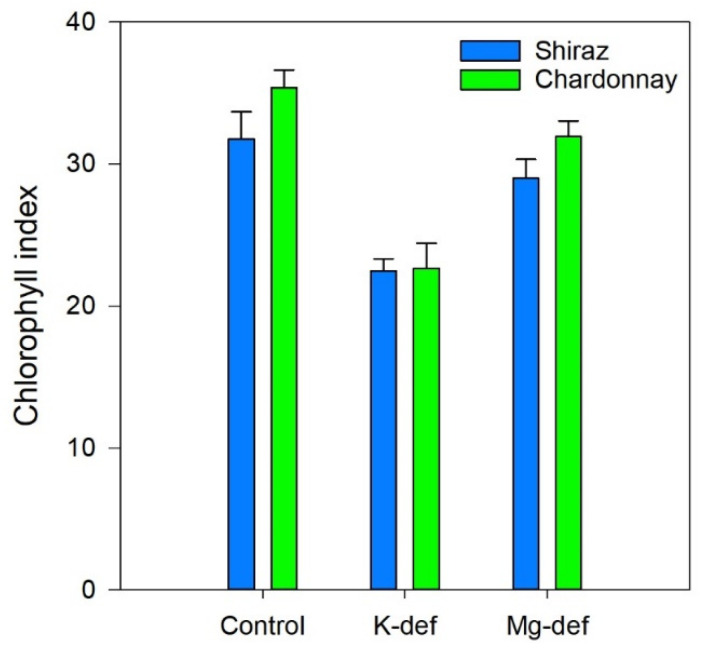
Chlorophyll index of Chardonnay and Shiraz vines after 3 months of hydroponic greenhouse conditions and grown in control, K-deficient and Mg-deficient nutrient solutions.

**Figure 7:**
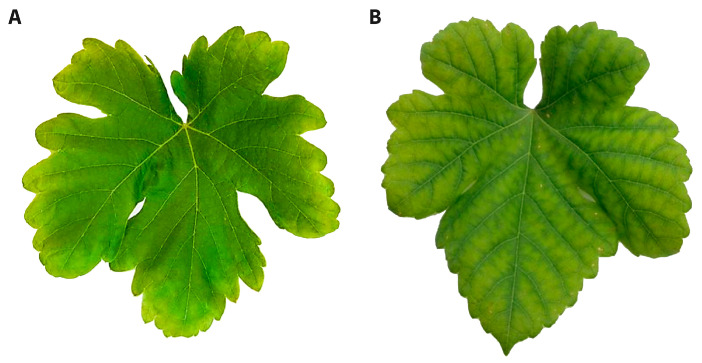
K (**A**) and Mg (**B**) deficiency symptoms on mature leaves of Shiraz vines grown hydroponically in greenhouse conditions.

In both cultivars, the leaf chlorophyll index was more strongly depressed in the K-deficient vines (30–35%) than the Mg-deficient vines (<10%) (Figure 6). Potassium deficiency resulted in chlorosis on the leaf margins, while Mg deficiency triggered interveinal chlorosis (Figure 7). Over time, necrotic areas formed on the margins (K) or in the leaf interior (Mg). No wilting was apparent.

### 3.6. Biomass Accumulation

**Figure 8:**
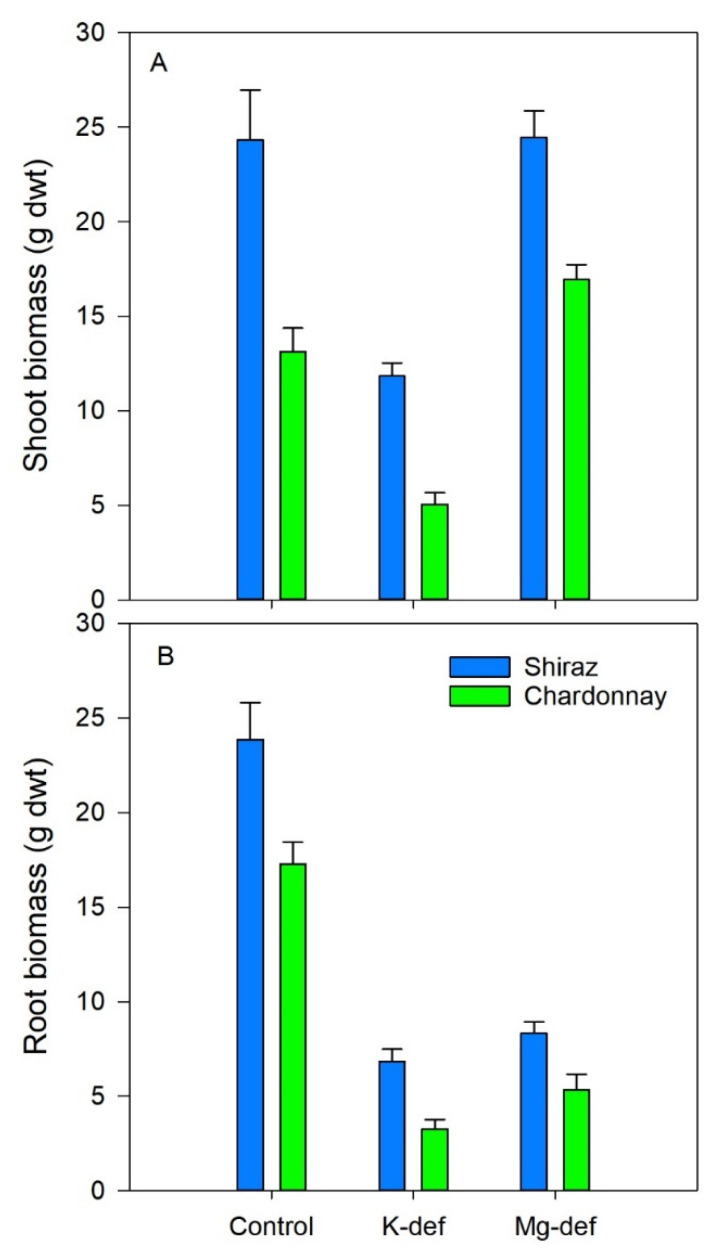
Shoot (**A**) and root (**B**) biomass (dry weight) of Chardonnay and Shiraz vines after 3 months of growth hydroponically in greenhouse conditions and grown in control, K-deficient and Mg-deficient solutions.

Biomass accumulation was curtailed in the deficient vines (Figure 8). Potassium deficiency reduced root growth by 70–80% and shoot growth by 50–60% in both cultivars (*p* < 0.001). Magnesium deficiency reduced root growth by 65–70% in both cultivars (*p* < 0.001) but shoot growth was not adversely affected in either cultivar. However, there were intrinsic cultivar differences, with Shiraz vines outperforming both root and shoot growth compared to the Chardonnay vines in all growth conditions.

## 4. Discussion

Magnesium and potassium deficiency markedly impaired photosynthesis in both Shiraz and Chardonnay vines when grown in these nutrient deficient conditions. This was evidenced by declines in *A*_400_ (photosynthesis at ambient CO_2_), *A_max_* (CO_2_-saturated photosynthesis) and *A_sat_* (PFD saturating photosynthesis). Reduced photosynthesis in response to K deficiency has been well reported in cotton [18], sugar beet [50], sugarcane [51] and tomato [52] while photosynthesis depreciation in Mg-deficient plants has been also observed in arabidopsis [34], sugar beet [53], beech [54], broad bean [55] and blue-green algae [56] among others. Thus, both K and Mg deficiencies induce widespread problems affecting productivity through inefficient harvest of solar energy thereby reducing photoassimilate supply.

In our vines, the severity of the decline in photosynthesis was dependent on leaf location along the shoot. The older and middle-aged fully expanded leaves of both cultivars were severely affected by the Mg deficiency. Potassium deficiency also reduced *A_net_* in older and middle-aged leaves of Chardonnay but in Shiraz vines, the older leaves were not as severely compromised. Those leaves that were still expanding and not fully mature were not affected by the nutrient deficiencies, suggesting that at least in Chardonnay both K and Mg were mobile and transported to the growing sinks from the older leaves or other storage sites in the vine. Other studies have shown similar mobilities for these nutrients [57]. This has important implications for reproductive growth, as flowers and berries can be a strong sink for nutrients through to maturity [58] at the expense of other growth points [59]. Our vines did not bear any inflorescences, and this is likely why the new leaves were favoured as sinks. The lower mobility of K in Shiraz may hinder partitioning to the crop, and thus inadequate ripening may be more prominent in this variety relative to Chardonnay. These vines were still young and, if the measurements had been carried out later in the season, a decline in *A* in the older leaves may have eventuated, as is common during normal aging and senescence [60]. Heavy shading of the internal canopy will also result in lower assimilation [61], but the basal leaves of our miniature vines with vertically trained shoots were well exposed to light.

The decline in net assimilation in response to Mg and K deficiency can, at least, be partly attributed to a reduced stomatal conductance (*g_s_*) in both Shiraz and Chardonnay vines. The decline in *g_s_* with K deficiency is well-documented [20,21,62], however, some studies have shown either no or the opposite stomatal response, and this may be related to the extent of K starvation or interspecific differences [22]. Lower *g_s_* in Mg-deficient pine seedlings was accompanied by reductions in the photochemical yield [63], indicating reductions in PSII photochemical performance, in keeping with the present results. In our vines, nutrient starvation not only decreased the stomatal elements of the photosynthetic response, but the non-stomatal attributes were also downgraded. The role of these attributes in CO_2_ fixation was clear. At low CO_2_ (less than 400 ppm), the carboxylation of RuBP limited assimilation, but the regeneration of RuBP was limiting at the higher CO_2_ concentrations.

In Chardonnay under Mg deficiency, there was co-limitation by RuBP carboxylation and regeneration at low CO_2_. The apparent maximum rates of RuBP carboxylation (*V_cmax_*) were also severely reduced in response to both nutrient deficiencies. Again, Chardonnay carboxylation was more sensitive to Mg deficiency than K deficiency, but Shiraz carboxylation was apparently equally sensitive to both nutrients. The apparent maximum rates of assimilate electron transport (*J_max_*) followed similar trends to *V_cmax_*, indicative of impeded RuBP regeneration by these nutrient deficiencies. It is well established that Mg modulates RuBP carboxylase [64,65]. Proton pumping from the stroma into the intrathylakoid space is counterbalanced by Mg^2+^ transport in the opposite direction [66,67]. *V_cmax_* and *J_max_* limitations in photosynthesis were also apparent in K-deficient rice [68] and hickory seedlings, more so than *g_m_* or *g_s_* [22].

Leaf chlorophyll content was apparently lower under Mg- and especially K-deficient vines, as evidenced by the chlorophyll index data as well as visual symptoms. Typical symptoms of K deficiency include chlorosis that initiates at the leaf margins, which then spreads inwards, while, in Mg deficiency, interveinal chlorosis is characteristic, and this may be exacerbated by high light through ROS destruction of chlorophyll and membranes [69]. Because sucrose export from leaves was inhibited by a deficiency in these elements [17], chlorophyll degradation may have occurred through feedback inhibition. Chlorophyll fluorescence is a well-accepted indicator of PSII function. Both cultivars had lower chlorophyll fluorescence attributes, including decreased efficiency of PSII photochemistry, increased photochemical quenching and decreased electron transport rates (ETR) under both nutrient deficiencies. Other studies have confirmed that Mg deficiency can impair PSII functioning [56] or both PSI and PSII photochemistry [53]. Magnesium is the central molecule of chlorophyll that absorbs photons and initiates electron flow [70]. Potassium- offers no structural purpose; however, it acts as a counter-ion to light-induced H^+^ flux across the thylakoid membrane [71]. It also establishes the trans-membrane pH gradient for the synthesis of ATP [30,72]. Consistent with this, these present data confirm the deleterious effects of these nutrient deficiencies on the photochemical and metabolic processes of photosynthesis in the leaves of the Shiraz and Chardonnay vines.

Dark respiration was higher in the nutrient-deficient Shiraz vines, although no differences were apparent in Chardonnay, perhaps indicative of increased metabolism to counteract the nutrient deficiencies, at least in Shiraz vines. Consistent with this, sunflower [73], spinach [74] and oil palm [75] have also showed higher respiration in K deficiency, while sugar beet had higher respiration in Mg deficiency [76]. Both growth and cellular maintenance contribute to respiration and, since growth was depressed under nutrient deficiency, it seems likely that maintenance respiration may have been upregulated. Considering that Mg is important to enzymes in glycolysis, the pentose phosphate pathway and the tricarboxylic acid cycle, we hypothesised its deficiency would have down-regulated respiration. Perhaps processes that compensate for the ion deficiency are upregulated in an attempt to maintain homeostasis, and these are not as energy-efficient.

Root growth of the Shiraz and Chardonnay vines was severely stunted in both K and Mg deficiencies. This was in contrast to N and P deficiencies, which favour root growth [77]. Curtailed phloem export from the leaves, in particular sucrose- and Mg-containing amino acids, may have contributed to the decline in root growth and root to shoot ratio [30,77]. The roots of K-deficient bean plants had lower sucrose and starch than those grown in adequate K [17], conforming to the observation that K is specifically required for phloem loading in grapevines [78]. Weakened root growth is typical for Mg deficiency [79] and, again, impairment of carbohydrate export from source to sink through slow ATPase-driven phloem loading of sucrose [72] may be one of the underlying factors contributing to reduced root growth. Shoot growth was compromised under K deficiency in both cultivars, and was probably a consequence of the restricted photosynthesis described here, but K also maintains the pressure potential required to drive cellular expansion [80]. Considering that K activates membrane bound proton-pumping ATPases [17] and is the main solute required in vacuoles for cell extension [81], reduced leaf and stem growth rates may explain the lower biomass of these vines. Potassium deficiency has also altered water relations in tomato, as evidenced by greater stem and fruit shrinkage, and the biomass of all organs was reduced [52].

Overall, the results suggest that both cultivars responded negatively to K and Mg deficiency, but Chardonnay may be somewhat more sensitive. In the field, Shiraz tends to have a more vigorous growth habitat than Chardonnay, and this agrees with our biomass results under all three treatments. The leaf morphology of grapevine cultivars is relatively plastic and highly sensitive to environment, and underlying biochemical changes such as anthocyanin production can occur in response to abiotic stresses [82]. Even though Chardonnay is a green-skinned cultivar, its leaf petioles are apt to be more reddish in appearance than that of Shiraz, which produces red berries. This was, however, not visibly amplified by the nutrient deficiencies and the laminas also did not have obvious differences in coloration. Further work is required to better understand the underlying biochemical differences driving the altered photochemistry in these two cultivars. The lower apparent sensitivity of Shiraz *g_s_* to nutrient deficiency may be related to this cultivar’s propensity to a more open stomata under optimal conditions. Previous studies have demonstrated that Shiraz leans towards anisohydry, with oscillating plant water status in response to fluctuations in soil moisture or evaporative demand [83], and an investigation of how water-stressed vines respond to nutrient deficiencies would be worthwhile. Shiraz also tends to ripen later than Chardonnay and berries undergo shriveling and senescence in warm climates once berries are mature [84]. Warm temperatures and drought [85] exacerbate the associated programmed cell death. Nutrient imbalances may also cause localized cell death and tissue necrosis [69] and the laminas of both cultivars ultimately suffered such symptoms despite any protective mechanisms that may have been in place.

## 5. Conclusions

The relationship between vine nutritional composition and vine functioning is often not evident for a specific nutrient. With our system, we have demonstrated that both K and Mg deficiency resulted in severe photosynthetic limitations, with consequent repercussions on above- and below- ground growth. Potassium and Mg starvation reduced stomatal conductance, but there were also biochemical limitations through both reduced Rubisco activity and RuBP regeneration, directly impairing assimilation. PSII functioning was also comprised, suggesting that some reduction in light interception occurred. This was in keeping with some apparent leaf chlorosis caused by the nutrient deficiencies. Because K is necessary for assimilate translocation, it is possible that feedback inhibition of sucrose accumulation in leaves also contributed to reduced photosynthesis. Given these results, and the known increased demand for assimilates by the reproductive system of grapevines [86], characterisation of the source-sink relationship in vines supporting bunch growth with these nutrients in deficit is warranted to achieve the goal of sustainable productivity in vineyards.

## Figures and Tables

**Table 1:** Magnesium and K content of Shiraz and Chardonnay petiole samples collected at the 10-leaf stage (g kg^−1^ dry weight) from grapevines grown hydroponically in greenhouse conditions.

	Shiraz		Chardonnay	
Treatment	Mg	K	Mg	K
Control	6.2	41.7	7.1	33.6
Mg deficiency	1.2	41.8	1.2	41.1
K deficiency	18.5	2.8	17.3	3.9

**Table 2:** Attributes of the curve fitting to photosynthetic light responses.

		Control	−K	−Mg	*p*
Shiraz	*A_max_* (µmol m^−2^ s^−1^)	11.02 ± 0.7	8.00 ± 0.08	8.17 ± 0.19	<0.001
	*ϕ_I_* (mol mol^−1^)	0.035 ± 0.000	0.030 ± 0.000	0.034 ± 0.001	<0.001
	*R_d_* (µmol m^−2^ s^−1^)	0.19 ± 0.01	0.47 ± 0.00	0.37 ± 0.03	<0.001
	*A_sat_*_(_µmol m^−2^ s^−1^)	840 ± 13	697 ± 5	637 ± 9	<0.001
Chard	*A_max_* (µmol m^−^^2^ s^−1^)	14.1 ± 0.3	7.0 ± 0.6	9.9 ± 0.4	<0.001
	*ϕ_I_* (mol mol^−1^)	0.039 ± 0.001	0.012 ± 0.002	0.020 ± 0.002	<0.001
	*R_d_* (µmol m^−2^ s^−1^)	0.081 ± 0.040	0.014 ± 0.001	0.35 ± 0.14	ns
	*A_sat_* (µmol m^−2^ s^−1^)	963 ± 26	1253 ± 53	1364 ± 103	<001

*A_max_* = light saturated photosynthesis, *ϕ_I_* = apparent photon yield, *R_d_* = dark respiration rate, *A_sat_* = photon flux density of light-saturated photosynthesis for leaves of Shiraz and Chardonnay vines grown hydroponically in greenhouse conditions.
